# Establishing the Geriatric Emergency Department Intervention in Queensland emergency departments: a qualitative implementation study using the i-PARIHS model

**DOI:** 10.1186/s12913-022-08081-4

**Published:** 2022-05-23

**Authors:** Marianne Wallis, Alison Craswell, Elizabeth Marsden, Andrea Taylor

**Affiliations:** 1 Faculty of Health, Southern Cross University, Southern Cross Drive, Bilinga, Queensland Australia; 2 Sunshine Coast Health Institute, Birtinya, Queensland Australia; 3 School of Nursing, Midwifery and Paramedicine, University of Sunshine Coast, Sippy Downs, Queensland Australia

**Keywords:** Geriatric, Emergency medical services, Implementation science, Hospital, Delivery of health care

## Abstract

**Background:**

Frail older adults require specific, targeted care and expedited shared decision making in the emergency department (ED) to prevent poor outcomes and minimise time spent in this chaotic environment. The Geriatric Emergency Department Intervention (GEDI) model was developed to help limit these undesirable consequences. This qualitative study aimed to explore the ways in which two hospital implementation sites implemented the structures and processes of the GEDI model and to examine the ways in which the i-PARIHS (innovation-Promoting Action on Research Implementation in Health Services) framework influenced the implementation.

**Methods:**

Using the i-PARIHS approach to implementation, the GEDI model was disseminated into two hospitals using a detailed implementation toolkit, external and internal facilitators and a structured program of support. Following implementation, interviews were conducted with a range of staff involved in the implementation at both sites to explore the implementation process used. Transcribed interviews were analysed for themes and sub-themes.

**Results:**

There were 31 interviews with clinicians involved in the implementation, conducted across two hospitals, including interviews with the two external facilitators. Major themes identified included: (i) elements of the GEDI model adopted or (ii) adapted by implementation sites and (iii) factors that affected the implementation of the GEDI model. Both sites adopted the model of care and there was general support for the GEDI approach to the management of frail older people in the ED. Both sites adapted the structure of the GEDI team and the expertise of the team members to suit their needs and resources. Elements such as service focus, funding, staff development and service evaluation were initially adopted but adaptation occurred over time. Resourcing and cost shifting issues at the implementation sites and at the site providing the external facilitators negatively impacted the facilitation process.

**Conclusions:**

The i-PARIHS framework provided a pragmatic approach to the implementation of the evidenced-based GEDI model. Passionate, driven clinicians ensured that successful implementation occurred despite unanticipated changes in context at both the implementation and host facilitator sites as well as the absence of sustained facilitation support.

**Supplementary Information:**

The online version contains supplementary material available at 10.1186/s12913-022-08081-4.

## Background

Emergency Departments (EDs) are chaotic environments in which frail older people, with complex medical problems, are placed at risk of iatrogenic complications as they are often subjected to prolonged ED lengths of stay and excessive tests [1–6]. Given the ageing of the population nationally, the importance of providing appropriate, high-quality health care throughout the ED and hospital journey, for this cohort, is paramount.

There are a variety of programs that focus on geriatric emergency care or hospital avoidance for older adults, in Australia [7, 8] and across the world [9]. More recently the Geriatric Emergency Department Intervention (GEDI) model was trialled in Queensland, Australia [10]. The GEDI model is a nurse-led, physician-championed model of ED care consisting of, but not limited to frontloaded geriatric tailored assessment, nurse-initiated specialist referral and shared decision making between the patient, any substitute decision makers and ED clinicians. GEDI is a service managed within the ED and is responsive to the needs and timelines of ED to facilitate appropriate referral and discharge planning. However, the GEDI model fundamentally incorporates a ‘border spanning’ role aimed at improving inter-disciplinary communication, entrenching patient-centred decision making, facilitating safe hospital discharge where possible and improving fast-tracking of referral and admission processes when required [11]. The GEDI team prioritise care for residents from aged care facilities and the frail older person [12].

Initially, the GEDI model was successfully implemented, in one ED in Queensland [10, 12]. A non-randomised trial indicated that, when the service was in place, ED and hospital lengths of stay, ED re-presentation rates and costs to the health service, were all reduced for adults aged 70 years and older [10]. The GEDI model was awarded the Queensland Premier’s Award for Excellence in Consumer Focus in 2016. Subsequently, a number of state-funded hospital EDs indicated a willingness to adopt the GEDI model and additional, non-recurrent funding was provided to these hospitals to implement the GEDI model. What was not clear was how well this model would translate into these other EDs.

Translation of research into practice can be fraught with difficulty [13, 14]. A full understanding of the difficulties associated with translating, adapting, integrating and diffusing evidence-based care innovation is still needed [15]. An implementation science project, using the i-PARIHS (innovation-Promoting Action on Research Implementation in Health Services) approach to implementation, was undertaken. This approach focuses on innovation, recipients of change and context and how these elements are impacted by facilitation [16]. The evaluative research study exploring the structures, processes and outcomes of this implementation included a quantitative study of the outcomes [17] while this article reports on the qualitative component of this project. The aims of this qualitative study were to explore the ways in which the implementation sites implemented the structures and processes of the GEDI model and to examine the ways in which the i-PARIHS approach influenced implementation. Additionally, we aimed to identify any aspects of the i-PARIHS framework that may need further expansion or consideration.

## Methods

The larger project employed a multi-method, multi-phase research design within the pragmatic paradigm. This philosophical paradigm allows the researcher to focus on “what works” and provides solutions for problems utilising methods that best meet their needs and purposes [18]. The part of the project reported here employed a descriptive qualitative method [19] and, as such, the Consolidated criteria for reporting qualitative research (COREQ) checklist [20] was used to ensure accurate and complete reporting of the study. As this was an implementation study the StaRI checklist [21] was also used to ensure accurate reporting of the implementation elements of the study. Staff from two hospitals were interviewed, nine to 15 months after implementation commenced. Hospital A was a large hospital located in the tropical north of Australia. Hospital B was a medium sized hospital west of Brisbane, Queensland Australia. The management of both hospitals had agreed to trial the implementation of the GEDI model in their respective EDs.

### Innovation to be translated

GEDI is a nurse-led, physician-championed innovative model of care that aims to improve outcomes for frail older persons presenting to the ED. The GEDI nurses are advanced practitioners who have additional experience and education in gerontology and care of frail, older people. They work with the primary care ED nurses and ED physicians providing targeted geriatric assessment, multi-disciplinary shared decision-making and coordination of care to facilitate rapid access and coordination of care through ED, hospital and community services. The details of this model of care are presented elsewhere [10, 12, 22]. Critical to the integration of the GEDI model into the ED is the role the ED physician plays in driving acceptance, policy change, and clinical support to overcome barriers to implementation. An extensive toolkit was developed, as part of this project, to assist in setting up and successfully employing this approach to care [23].

### The approach to knowledge translation used in this study

The implementation of the GEDI model in two Queensland EDs was based on the i-PARIHS approach to facilitation [16] and a Cochrane review that provides evidence of the importance of tailoring interventions to the context [24]. The i-PARIHS framework rationalises implementation of research into the clinical practice settings through implementation context, facilitation as integral to success and the recipients of change [16]. As a first step the project team developed a GEDI Implementation Toolkit [23] which was tailored to the state government policies and procedures. In line with the i-PARIHS model, the implementation process was then managed procedurally by two layers of facilitators, external and internal. The external facilitators (EFs) consisted of the ED physician and senior GEDI nurse who had been involved in the initial GEDI trial. According to i-PARIHS, the EF is a knowledge broker, linking the knowledge producers (i.e., clinical and research team) to the recipients or knowledge users (i.e., hospital ED staff) [16]. In this project these EF clinicians had skills in knowledge translation, change management, negotiation and influencing, and their activities included mentoring, coaching and guiding the internal facilitators. They also developed resources related to facilitation. These resources included a web-based toolkit, and short video vignettes for use by the internal facilitators where ‘tricks of the trade’ were shared.

**Fig. 1:**
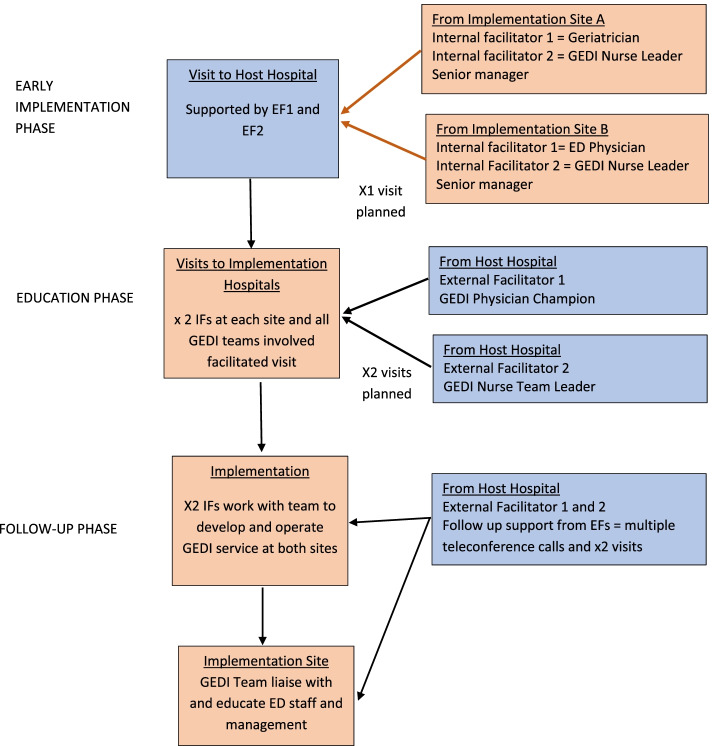
Diagram of Planned Implementation Strategy

It was planned that these two EF clinicians would host visits, for the internal facilitators (IFs), at the ED where the GEDI model was successfully implemented and visit both new implementation sites a number of times before and during the implementation. They would then have regular telephone or videoconference calls with the implementation sites (see Fig. 1). Funding from the Corporate Division of Queensland Health, the state-wide public hospital and health service provider, was given to their public hospital to release them from other duties and allow them to engage fully in supporting the implementation sites. At each implementation site there were two IFs who were the local champions. The IFs (ED physicians and senior nurses at each site) reflect the role of boundary spanners in i-PARIHS [16]. In this approach to implementation the IFs interact and connect with local staff and the EFs (See Fig. 1).

The IFs bring content expertise, related to care of older adults in the ED, contextual knowledge of the hospital system and how to navigate local hospital processes. It was planned that the IFs would receive guidance from the EFs to develop and apply skills in knowledge translation and change management. The IFs were the expert clinicians who would manage the program. It was planned that the IFs would present the toolkit to staff and establish a local support program, enabling local staff to share their learning about what worked and what did not work in the local context. They would also work with other GEDI and ED staff to undertake an environmental scan and then develop an action plan to maximise enablers and overcome barriers to implementing GEDI [25].

### Evaluation of GEDI implementation

Semi-structured, audio recorded, interviews were conducted with a range of staff at the two implementation sites, by author AC, who has PhD and post-doctoral training in qualitative interviewing. The author AC was not known to the interviewees, prior to the interviews but had worked as the Research Fellow/Project Manager on the original study evaluating the GEDI model.

A purposive sample of relevant medical and nursing staff was contacted via email. Emails inviting staff to participate in the study were sent to the IFs and EFs, middle managers in ED involved in the implementation, individuals who were appointed to GEDI roles and frontline ED clinicians. Study information was sent out by the administrative assistant of the ED. Once individuals had indicated a willingness to be interviewed, they were contacted by the interviewer. Informed consent was obtained from interested staff and interviews were scheduled at a time and place suitable to that staff member. All staff members elected to be interviewed in a private space in their workplace and all interviews took between 15 and 60 minutes.

The interviewer employed a range of different questions and prompts for each of the groups of participants. Each participant was asked to explain their role and how they were involved in or interacted with the GEDI model. Then participants from each group were asked to reflect on the implementation process, how the GEDI model impacted their role and how it influenced workplace practices in the ED. Please see Additional File 1 (Supplementary Materials) for a copy of the interview schedule.

### Data analysis

Transcribed interviews were read and re-read, and an initial label (code) was assigned to sections of text relating to the adoption or adaptation of different aspects of the GEDI model by the sites; and the factors influencing implementation at each site. Codes were then amalgamated into categories and themes [26]. This initial coding was undertaken by MW. This researcher is a very experienced researcher who has both doctoral and post-doctoral training and experience in qualitative research methods and has taught these methods for over 30 years.

The i-PARIHS framework conceptualises successful implementation (SI) as involving facilitation (Fac^n^) that addresses the innovation in practice (I), the recipients of the innovation (R), and the quality of the context (C) [SI = Fac^n^(I + R + C)] [27]. Consequently, in addition to the coding and theme development described above, the qualitative data were explored in terms of innovation (and evidence), recipients and context and how these elements were impacted by facilitation.

Once the initial analysis had been undertaken by MW and illustrative quotes had been presented in support of the sub-themes and themes, all authors discussed the findings. As EM and AT were the external facilitators and thus heavily invested in the project, the other authors MW and AC ensured that any emergent themes that were challenged by EM and AT could be supported by interview data from study participants. The study findings were not able to be returned to participants as most participants had left their positions and were not contactable once analysis was undertaken.

## Results

In total 17 interviews were recorded with staff in Hospital A and 12 in Hospital B, in addition the two external facilitators were also interviewed. These data provided theoretical saturation along with deep and rich descriptions of the barriers and enablers to implementation and the utility of the i-PARIHS approach to implementation. The roles of the people interviewed included middle managers and senior GEDI staff involved in setting up the model of care, middle managers involved in the on-going management of the model of care, GEDI clinicians (medical, nursing and pharmacy), ED clinical staff and the external and internal facilitators involved in the implementation process.

Generally, all interviewees expressed support for the GEDI model, and the toolkit developed during the initial research. A key function of the GEDI team is to support the ED primary care team to make decisions about whether a frail, older adult needs to be admitted to the hospital, or whether they can be discharged safely home, and access care in the community. As an ED nurse said,

*“I think (the GEDI model) helps flow... Either it’s, “You’re probably going to be discharged. GEDI have already been in and worked that out. Your daughter is on the way.” That happens quickly, or what I’ll see is GEDI have come in and found a problem, and had it looked at and realised (going) home’s not going to work. They’re going to need a referral and (…) that happens earlier as a result of more investigation or more history taking on their part. And I think that definitely (improves) flow because we’ll arrive at that decision much earlier to refer rather than discharge.”* (ED Nurse: Hospital A).

Analysis of the data identified three major themes. There were elements of the GEDI model (as detailed in the Toolkit) that had either been: (i) adopted or had been (ii) adapted by each site and altered in ways not suggested by the Toolkit. As one GEDI CNC said, ‘*Yep, great we’ve got the GEDI; but you could adapt it.’* (GEDI Nurse: Hospital B). In addition, there were data that related to the (iii) factors that affected the implementation of the GEDI model.

### Adoption and adaptation of the GEDI model

There were five sub-themes, each with a number of categories, identified that related to the elements of the GEDI model that were adopted and/or adapted for use in the study EDs. These categories were: Team Structure, Service Focus, Organisation and Funding of the GEDI model, Staff Education and Data Collection for Service Evaluation. Table 1 provides these sub-themes and categories along with exemplar quotes from the study participants.

**Table 1:** Sub-themes, categories and exemplar verbatim quotes related to the two major themes of adoption and adaptation

Adoption		Adaptation	
Sub-themes and Categories	Exemplar quotes	Sub-themes and Categories	Exemplar quotes
**Team structure**			
GEDI nursing team have extensive experience in community care for older adults and have some experience working in ED.	“One of the Nurses has a lot of community experience and as far as I know, has quite a good scope with aged care. And the other two had a keen interest.” (ED NUM: Hosp A). “I: Have you had any other prior experience with geriatrics other than in the ED? R: Yeah, I’ve worked as a nurse for over 20 years now, on the wards and in the community”. (GEDI CNC: Hosp B).	Team member background - not community or/ gerontology nurses rather ED nurses with training	“Going on (the external facilitators’) advice it was hard to get the trifecta of having (gerontology), ED and community experience, and knowing what services are available. You know… that medical experience and then that ED experience”. I: All of your GEDIs were ED staff before? R: Yes. So, two of them were ED staff and one was a casual Nurse who only worked in ED.” (ND ED: Hosp A).
Physician champion is ED physician with additional training in gerontology	“I: And so, when did you sort of get the physician champion? R: They were all brought on straightaway” (ED SMO: Hosp B)	Physician champion role varied and seen as change champion only	“We had quite a difficult start with GEDI. We felt a little bit unsupported in, not the first few months but when we got into it. It was just because our Champions got sidestepped into other roles, so we were left a little bit”. (GEDI CN: Hosp A)
		Included geriatrician and allied health roles in GEDI	“The first three weeks it was just (the GEDI CNC), then we had the physio and the geriatricians and the CN, a full time CN come on board.” (GEDI CNC: Hosp B5) “(As the GEDI Pharmacist) I stay in ED. When GEDI patients leave ED, and I’ve seen them, they’ve got a medication action plan. If there’s any major concerns or anything like that, I’ll often call the ward Pharmacist. If they’re going home, I’d write the letter to the GP expressing my concerns etcetera.” (GEDI Pharmacist: Hosp A) “it’s been great having a pharmacist. (The GEDI Pharmacist) is great at educating you. He’s (also) really proactive and he gets in there so quickly. He does call the GP, he’ll source that from here and he knows how to access, where it’s really helpful for the doctors as well as the patients. He does expediate a lot of the little hurdles that come along.” (GEDI CN: Hosp B)
**Service Focus**			
GEDI Nurse focused on disposition decision making	“I can picture 100 times where (GEDI say to) ED Doctors, ‘Actually, no. I’m not sure that we can (…) send them home.’ Or ‘Maybe short stay isn’t appropriate; they do need an in-patient admission’. And they’re listened to and respected (…). So, a good part of the team”. (ED Nurse: Hosp A) “It was really, really good especially if you went with a firm plan (and said) ‘I really want to refer on to the memory clinic at some point, but I need your signature. Are you okay with that?’” The Doctors just worked with me. They were fine. (GEDI CN: Hosp A)	GEDI nurses did some of the clinical care the primary nurses didn’t have time for.	“So, you know if we still have to, we’ll still do the bloods, the cannulas, the ECGs. And we’re not doing it so much as to do the primary nursing, we’re doing it to expediate getting those patients results and that kind of thing.” (GEDI CN: Hosp A) “…when I spoke to her about it, she said, “Oh, the team leader in ED asked me to do it”. She was taking bloods from port-a-caths and you know, doing the general pathology, taking ECGs, which I’ve done, we can all do, but I didn’t want us getting into that role so early on.” (GEDI CNC: Hosp B).
**Organisation and funding of GEDI service**			
Hours of service = 7 days and weekdays 0730–1930. Weekends 0730–1600	“They’ve got quite good hours, coverage is quite good.” (Director EM: Hosp A)	Shifted GEDI focus away from RACF and frail older adults to people admitted post fall	“…if it’s not working in its current form, why can’t we adapt it a little bit and look a little bit more towards our falls and our geriatric speciality in the ED department. Because it doesn’t have to be hard and fast, does it? So, we could actually adapt the model and maybe that’s what is needed so that people know exactly where they’re going.” (CNC Dementia: Hosp A)
Funding of positions from ED budget	“…it (the funding) runs out the end of December, but I gather there may be more money in the offering. But I know nothing in terms of whether that’s confirmed…” (ND: Hosp A)	Funding may not be available after the trial	“I’m not sure that there is capacity within the financial situation of the HHS to fund anything above what is currently funded. I think even with demonstrated benefits of financial savings and support (…). I think there’s a good level of knowledge of the benefits for GEDI (…). The trouble is it doesn’t actually save money if someone stays in that bed if you put someone else into the bed, unless you close the bed behind the person (…).” (Geriatrician: Hosp A).
**Staff education about GEDI role and care of frail older adults**			
GEDI nurses need specialist education about gerontology and ED nursing	“… (a new GEDI CN) has said she’s just starting the UTAS course (Understanding Dementia MOOC).” (GEDI CN: Hosp A)	Staff education – GEDI staff insufficiently prepared for role	“…a patient lived in a nursing home, (and) had a fall. And (the new GEDI CN) recommended, (that) the patient be transferred to the rehabilitation unit which given the extent of the patient’s history and how long they’ve been in a nursing home, it really wouldn’t have been any benefit. But she wasn’t looking at the long-term benefit for the patient, she’d written that as an antidote to everything else about pathology and X-rays...” (GEDI CNC: Hosp B)
New staff need to be oriented to the role of GEDI in the ED and GEDI engage in staff development related to gerontology	“(The GEDI CNC)‘s done quite a number of in-services. I mean, initially when it was all set up we had a lot of education about what the role was and what was expected. And since then (…) I remember going to 4AT assessment, a delirium and dementia screening, (…) psych geriatric stuff. (…) And I know they’re asked to do them regularly on GEDI specific topics because we have a half hour in-service every day of two different groups.” (ED CN: Hosp A)	Staff education – ED staff only educated about GEDI role initially – GEDI role not included in new staff orientation	“I think there was some of that early on when the role first started, so there was certainly some education early on in the piece. It was mainly sort of targeted at the senior nursing staff, senior medical staff. Our junior staff tend to sort of rotate quite frequently, so keeping them up to date with initiatives like this is quite difficult. But again, because they’re so visible and so interactive with the patient there’s a lot of it on the floor, training round, “Who’s that? What’s GEDI?” So, there’s a lot of on the floor type training and that seems to work well”. (Director EM: Hosp A)
**Data collection for service evaluation**			
Data collection of ED outcomes and occasions of service	“…I did a lot of data in that (first) five months…” (GEDI Physician Champion: Hosp A)	Clinicians in GEDI roles may not have the skills to access and analyse clinical service data	“… to get that information together, but I was very bad at gaining that, understanding of what I was looking at.” (B/GEDI CNC/5). “It’s been a challenge finding that documentation sometimes in IEMR if it’s not gone through and onto the viewer and stuff. That’s one of the big challenges. And obviously it’s a very small FTE of staff working the role, so I’m having to (…) collect the data. It’s a big job. (ND ED: Hosp A)

Legend: *CN* Clinical Nurse (first level of specialty nurse in state system), *CNC* Clinical Nurse Consultant (second level of specialty nurse in state system), *ED* Emergency Department, *EM* Emergency Medicine, *GEDI* Geriatric Emergency Department Intervention, *Hosp A/B* Hospital A/B, *I* interviewer, *ND* Nursing Director (manager of hospital division comprising multiple units), *NUM* Nurse Unit Manager (manager of individual unit), *R* Respondent (interviewee), *SMO* Senior Medical Officer

The GEDI model specified a team approach, in which nurses with expertise in the management of frail older adults, especially in community settings, were upskilled in ED nursing and assisted the ED teams to make disposition decisions. These GEDI nurses were supported by a Physician Champion. This senior medical officer had additional training in the care of older adults and medical management of geriatric syndromes. This championing role extended beyond the change management process and meant that the GEDI model had a champion in senior medical forums and in management decision making and budget meetings. While the study sites, initially, understood why these roles were set up this way they all adapted the model. Most of the nurses were ED nurses who expressed an interest in caring for older adults but were expected to upgrade their knowledge and skills in their own time, using their own resources. Sometimes this meant they reverted to the role of providing nursing care in the ED and were diverted from assisting with disposition decision making. In addition, the implementation sites added geriatricians, physiotherapists and/or pharmacists to the team.

The Implementation project was structured around the i-PARIHS model with EFs and IFs, a structured program of visits/teleconferences by the staff from implementation sites to the development (host) site and vice versa and funding to support these activities. As can be seen from Table 2, what was planned (see Fig. 1) did not eventuate as the senior ED management in the host hospital felt they could not support releasing the EFs to allow them to facilitate at the implementation sites. The differences between what was adopted from the model and what was adapted were sometimes seen to be really useful e.g., adding a pharmacist or physiotherapist to the team. Other adaptations were less successful e.g. employing ED nurses with no experience of community/gerontology nursing as GEDI nurses. The breakdown in the implementation plan meant that adaptions were not challenged and the evidence for changes to the model was not established. However, as one participant put it, *“I think actually since this program started it has highlighted to other (ED) staff, (…) that we should be learning more about dementia. (…) There was a dementia/delirium workshop recently and a lot of them applied for it because they’re interested in learning more. (…) It’s a good thing. It’s really highlighted how we wish we could and should be doing a lot better for geriatrics in emergency departments.”* (ED NUM: Hosp A/1).

**Table 2:** Themes, categories and exemplar verbatim quotes related to the implementation process

Themes Categories	Implementation		
	What was planned	What was implemented	Supporting quote
**Planning**			
Evidence-based model	GEDI model – based on the GEDI Toolkit – structure, process and outcome evaluations published [11–13, 22]	A dedicated team operating 12-hour days a week and 8 hours at weekends not necessarily comprised of the same workforce as original GEDI model	“GEDI needed a physio because the majority of the patients present with falls, or they’ve fractured something, or just they’ve got pains in the knees. So, we funded for a physio.” [Hosp B: Int 5_] “(The GEDI service is) staffed by nursing and pharmacy and with the intention of, if possible, reducing the number of elderly patients that get admitted to hospital.” [Director EM: Hosp A].
Collaboration between clinicians and managers	Managers fully supportive of GEDI implementation, advocating for GEDI with Executive.	Supportive managers moved, new managers not aware of extensive background for model.	“Initially, one of our (ED physicians) that’s his portfolio, is aged care. So, he definitely gained a lot of support” (ED NUM: Hosp A] “The only thing that got this lot moving was the CE (Hospital Chief Executive).” [GEDI CNC: Hosp B] “I do feel that there’s not the support there anymore.” [GEDI CNC: Hosp A]
Governance	GEDI team to be managed by ED managers with GEDI senior nurse part of ED management team. Physician champion involved in day-to-day management and support of GEDI nurses.	Physician champion involved in initial change management – then transferred. GEDI seen as primary care ED nurses with additional older adult focus.	“…our Champions got sidestepped into other roles, so we were left a little bit. “[GEDI CN: Hosp A]. “… (an ED nurse who took on the role of a GEDI CN) seemed really keen and really interested, but with every patient she reverted back to being an ED nurse. She saw the patient and she was doing the bloods, ECGs…” [GEDI CNC: Hosp B]
**Funding**			
Funds for i-PARIHS implementation	Funding provided to backfill staff to be involved in GEDI model facilitation and for travel and accommodation to support site visits.	Funds transferred from Health Department to original GEDI site. Used to fund only 25% of planned implementation activities.	“We were just told we would not be able to be released to support (Hospitals A and B). Nothing we said changed her mind” (External Facilitator B).
Accountability for acquittal of project funds	Management at original GEDI test site were responsible for funds transferred from Health Dept. for implementation project.	Funding was managed in ED operational budget and not used to support EF release to engage in implementation site support.	“The funds were transferred to the ED operational budget from (State Health Department). No-one had to report back to State Health Department)” (External Facilitator B).
Recurrent funding of GEDI model	Senior staff at implementation sites to develop business plan	Managers changed roles. GEDI senior nurse asked to develop Business Plan	“So, (the Health Dept.) is set up for innovation for? 12 months and then we have to pick up recurrent funding. All I know is (physician champion) was putting in a business case to apply for funding to continue the program but I don’t know where we’re at.” (ED NUM: Hosp A) “I’m not sure that there is capacity within the financial situation of the (hospital) to fund anything above what is currently funded. I think even with demonstrated benefits of financial savings and support, I’m not sure how many services will get additional funding over baseline at the moment. I think there’s a good level of knowledge of the benefits for GEDI” (Geriatrician: Hosp A)
**Teams**			
Support of internal facilitators by external facilitators	• Having the External Facilitators (EFs) attend the implementation sites 2–3 times. • Have GEDI Implementation internal facilitators visit main GEDI site • Have weekly then monthly post-implementation meetings. • Have external facilitators available during service hours for consultation by telephone. (From interviews with external facilitators)	• EF B visited the two sites once. EF A visited one site once. • Some staff – mainly managers visited host site once • Four meetings with site A over 3 months EF A only able to attend one meeting • One visit to host site by Hosp B. No meetings. • Phone consultations between GEDI CNC at host hospital and both implementation sites for six months as needed. • No contact between physician champions after visits.	“I think the disappointing thing for me (…), is we were hoping (Hospital providing the external facilitators) would release (Physician Champion) and (GEDI CNC) for a day a month. Because their energy is infectious. I can sell it as much as I want but to have them in the room, you know how lively (External Facilitator A) is. (…) We hit a bit of a political barrier (…). (GEDI CNC: Hosp A) “So, we went down there for about three days or four days in January when it first started, and then (Dept. of Health staff member) and (External Facilitator B) came up here when we first implemented it and spent about three days here with us. And then I’ve been in a meeting with a couple of teleconferences since then, but not for months”. GEDI CN: Hosp A) “I: …have you had any other contact with (the EFs) since championing the role here in the ED? R: Not since the meetings were stopped, so I’ve only met them once.” (GEDI Physician champion: Hosp B)
Middle management support for GEDI model	Middle managers to continue enthusiastic support for GEDI model development and evaluation.	Managers changed. New managers did not see relevance of model in times of fiscal constraint.	“I (ED NUM) found it interesting and thought this is a good program we’d like to take on. So, (ED ND) and I both went down there to that, along with I think (middle manager) may have come as well. I can’t remember.” (ED NUM: Hosp A) “…we hit a bit of a political barrier” (ED ND: Hosp A)
Role of physician champion	A senior medical officer would act as a boundary spanner working with the senior GEDI nurse to develop business case, recruit and support GEDI nursing team. Also, would engage ED physicians in working collaboratively with GEDI nurses and supply decision support to GEDI nurses.	In one implementation site the ED physician was only involved during the initial change process. The decision support changed to a geriatrician not employed within ED. When this geriatrician changed jobs, the new geriatrician did not continue to support the GEDI team in the same way.	“(I wasn’t) directly involved in (setting up the GEDI service) but when I heard that it was the geriatricians who were interested in getting involved, I was happy to be the champion in my department. (GEDI physician champion: Hosp B) “(The GEDI geriatrician is) very particular on what patients they see… (The first GEDI geriatrician) did a role (description for the geriatrician (role) and it says (the GEDI geriatrician is) mainly for patients with the geriatric syndromes” (GEDI CNC: Hosp B)
**Toolkit**			
Toolkit utility	Written Toolkit provided as a pdf document that included (i) Background, rationale and evidence for GEDI, (ii) Explanation of the GEDI model, (iii) Resources to assist in setting up a GEDI service	Written toolkit available as a pdf document. Also, an online version and additional video vignettes explaining aspects of the model.	“It’s very long. It’s probably too long I’d say. (But) its comprehensiveness is good and it’s useful if you have a specific question. (…) So, if you use it like a sort of like a reference text. Then, yes. It was useful.” (ED Physician: Hosp A).

Legend: *CN* Clinical Nurse (first level of specialty nurse in state system), *CNC* Clinical Nurse Consultant (second level of specialty nurse in state system), *ED* Emergency Department, *EM* Emergency Medicine, *GEDI* Geriatric Emergency Department Intervention, *Hosp A/B* Hospital A/B, *ND* Nursing Director (manager of hospital division comprising multiple units), *NUM* Nurse Unit Manager (manager of individual unit)

## Discussion

The results of this qualitative study of the structures and processes involved in the implementation of the GEDI model and the outcomes study (reported elsewhere) [17] indicate that there was a successful implementation of a new model of ED care for older adults at the study sites. Localisation of the model occurred with varying degrees of adoption of recommended practice and local adaptation of structures and processes. The results of the quantitative study [17] suggest that the implementation process resulted in improved outcomes for frail older adults but not of the same quantum as demonstrated at the original trial site.

The evidence, for practical and successful ED intervention implementation, remains scant [28]. Projects in which interventions were implemented across multiple sites tailored i-PARIHS to suit their needs [29–33]. The results of their implementations had several common findings with our study including variation in model concordance [30], lack of accountability for tasks impacting workload [32], and a lack of protected time for implementation facilitation [33]. Evidence for implementation evaluation relating to care coordination for older adults also supports the findings of our study, specifically, that organisational involvement is critical, yet hampered by organisational change [31].

One of the key activities in successful implementations of health interventions is engagement that is early, continuous and widespread [34]. Experience with the initial implementation and trial of the GEDI model identified that engagement with the GEDI team, primary ED clinicians, and hospital management was required throughout the development process [35]. Key clinicians (recipients in the i-PARIHS model [16]) were active in requesting GEDI model implementation and were in contact with the model developers early in planning for implementation evaluation. This early enthusiasm for the model facilitated the beginning of the implementation process. However, continued engagement, between EFs and IFs, as required by the i-PARIHS model, was not sustained.

In this study, the initial evidence driving the innovation was high quality research that had been undertaken in a hospital very similar to, and in the same Australian state, as the two implementation sites [10, 12]. The recipient clinicians were very enthusiastic to adopt this change to their practice. The context, however, was negatively impacted by (recipient) staff changes at the implementation sites which meant key supports were lost. This reflects the real world of implementation and change in health services and is seen in other models internationally [36]. In addition, while the facilitation was well planned and resourced it was undermined by unforeseen local barriers at the site providing the EFs. A key element that was not sufficiently managed in this project was the context at the site providing the EFs. Having been provided with funding by the State Health Department, to release the EFs from their other duties, management at the original development site reversed their original commitment and refused to allow the EFs to engage in on-going facilitation and support of the recipient sites.

The i-PARIHS model [16] addresses the issues that might influence the outer context (e.g., organisational and external health system issues) for the recipients of change but in this study, it was the outer context of the site providing the EFs that was the larger issue. When using the i-PARIHS approach it may be useful to consider the outer context of all organisations involved in the facilitation model. Despite these issues, the GEDI implementation process adapted to these destabilising influences and successfully made changes to practice that the results of subsequent research showed improved patient outcomes, at the study sites [17].

We would contend that the i-PARIHS framework worked as well as any of the other possible frameworks we could have used (e.g., Knowledge to Action framework [37]), because the factor that most impacted consistency of implementation, organisational context, changed over the life of the project. We did not use a validated instrument to measure organisational commitment such as Organizational Readiness to Change Assessment instrument (ORCA) [38] but, had we done so, it would not have alerted us to any issues not already recognised.

The importance of middle management in practice change cannot be understated [39]. In this project, management staffing changes meant that support for the project was lost and there were no mechanisms within the State Health Department, nor the local hospital and health service, to ensure continuity of decision making and support for the model. The results of this study support the contention that middle managers occupy a significant role in health service organisations and their leadership training needs to have a clear focus on organization-level determinants of system change [39].

Finally, there was inadequate fiscal accountability. Having transferred funds from the state government to the hospital there was no requirement for the hospital to provide an accurate acquittal for those funds, they were just absorbed into the operating budget. These issues with financing new models of care for older adults in ED are not unique to Australia and resonate with similar research from the USA [40]. Middle manager responsibility for system change and service renewal should always be linked to fiscal responsibility.

### Limitations

For the qualitative interviews, all undertaken by one person, all the relevant people involved in the implementation phase were included, until data saturation occurred, in line with qualitative methods. Comments in the Results, however, indicated that following the exit of some staff the implementation process changed for those remaining and for those taking over from them. This was only a small number of people over the 12 months of the study but may have influenced the results. This is a known issue in the sustainability of change [41]. In addition, while every effort was made to ensure the data analysis was a true representation of the implementation, the inclusion of the facilitators in data analysis may have had unknown impact on the overall outcomes.

## Conclusion

While lack of transparency related to management decision making and an absence of fiscal accountability persist, effective implementation of evidence-based models of care will always be subject to changes in context. In this instance, implementation was successful due to passionate ‘grass roots’ ED clinicians driving a change in the model of care for older adults in ED. Success transpired despite the lack of some important aspects of the i-PARIHS model, such as, consistent middle management support at the sites that provided both the internal and external facilitation.

## Supplementary Information

**Additional file 1.**

## Data Availability

Data are available on request due to privacy or other restrictions: The data that support the findings of this study are available on request from the corresponding author. The data are not publicly available due to ethics restrictions and them containing information that could compromise research participant privacy/consent.

## Abbreviations

ACE: Acute Care of the Elderly
ASET nurses: Aged Care Services Emergency Team nurses
CARE-PACT: Comprehensive Aged Residents Emergency and Partners in Assessment, Care and Treatment
CN: Clinical Nurse (first level of specialty nurse in state system)
CNC: Clinical Nurse Consultant (second level of specialty nurse in state system)
ED: Emergency Department
EF: External facilitator (in i-PARIHS model)
EM: Emergency Medicine
GEDI: Geriatric Emergency Department Intervention
HINH: Hospital in The Nursing Home
I: interviewer
i-PARIHS: innovation-Promoting Action on Research Implementation in Health Services
IF: Internal facilitator (in i-PARIHS model)
ND: Nursing Director (manager of hospital division comprising multiple units)
NUM: Nurse Unit Manager (manager of individual unit)
R: Respondent (interviewee)
RACF: residential aged care facility
