# Supplementary material for: Establishing the Geriatric Emergency Department Intervention in Queensland emergency departments: a qualitative implementation study using the i-PARIHS model
Source: BMC Health Serv Res. 2022 May 23;22:692. doi: 10.1186/s12913-022-08081-4 (PMC9128293; doi:10.1186/s12913-022-08081-4)
Supplement: Supplementary file 1 — Additional file 1. [file 12913_2022_8081_MOESM1_ESM.pdf]

GEDI Implementation Study Interview Schedule

NB: Staff will be recruited as described in the National Ethics Application Form: via emails sent internally by the GEDI CNC, ED NUM and ED Medical Director. Additionally, a research team member will attend staff meetings and information sheets will be left in internal mail for all ED staff.
Fully informed consent will be obtained prior to interview.

Questions for general ED staff (nurses, doctors and allied health staff)

Questions:

1. What is your role here in ED?
2. How long have you worked here at NGH ED?
3. Have you seen the staff in the pink shirts? Do you know what service they provide?
4. What is your understanding of the GEDI role?
5. Do you interact with GEDI in your role?
6. Can you tell me about that?
7. In what ways if any, does the GEDI role impact on your role or the care you provide in ED?
8. How do you think the GEDI role impacts on patients in ED?
9. Have you identified any issues that need to be addressed in how the GEDI's fulfil their role?
10. Can you think of any way in which the GEDI service could be improved?
11. Is there anything else you would like to add?

Questions for GEDI Clinical Nurses, Clinical Nurse Consultant and Physician Champion

1. Could you describe the service setting you work in. How does this impact on your provision of service?
2. What impact if any does ED staffing have on the service you perform?
    a. GEDI staffing (i.e. sick leave)
3. What are the key resources (physical, other HPs etc) you require to carry out your service?
4. How accessible are these physical/staffing resources to you for carrying out your role?
5. Were there any barriers to the process of setting up the GEDI service? If so, can you describe them? Were you able to overcome them?
6. What are the issues around funding GEDI
7. Describe the process of an average day as a member of the GEDI team
    a. How are patients referred to GEDI
    b. What happens after hours?
    c. Can you list the key features of your role please?
    d. What is the process of communication with ED staff, with other HPs outside of ED?
    e. Could you describe what pathways are in place and if any have been developed since the GEDI role commenced – ie ortho-geriatric pathway etc
    f. What is your role in advanced care planning – how often are you involved?

    g. Describe any educational activities you are involved in.

8. Can you suggest how your role might be improved?
9. What policy and procedures have been put in place around the GEDI role?
10. How do you engage with GPs/Family Physicians, Residential Aged Care Facility staff?
11. How have GEDI influenced the care provided to patients since GEDI have been in the ED? (Prompts-: changes standard of care? Other ED nurse's skills/practice changes or changes decision making processes? Interest in Geriatrics?)
12. What other duties could a person in your position undertake if management issues or cost was not a factor?
13. Describe how you think practices have changed since GEDI started in ED – (can you describe the process for a patient before GEDI?)
14. Implementation? How has that worked for you? Prompts:
    a. Trip to initial implementation site
    b. Phone calls
    c. On site visits
15. If you were going to be involved in implementing a GEDI service again, what would you do differently?
